# A rare case of posterior mediastinal myelolipoma resected using robot-assisted thoracic surgery

**DOI:** 10.1016/j.ijscr.2025.111122

**Published:** 2025-03-04

**Authors:** Kiyoshi Sato, Yuki Shindo, Katsushi Toyohara, Satoshi Fumimoto, Nobuharu Hanaoka, Takahiro Katsumata

**Affiliations:** a Department of Thoracic and Cardiovascular Surgery, Osaka Medical and Pharmaceutical University Hospital, Takatsuki, Japan; b Department of Thoracic Surgery, Takatsuki Red Cross Hospital, Takatsuki, Japan; c Department of Thoracic Surgery, Hirakata City Hospital, Hirakata, Japan

**Keywords:** Myelolipoma, Mediastinal tumor, Posterior mediastinal myelolipoma, Robot-assisted thoracic surgery

## Abstract

**Introduction:**

Myelolipomas are rare tumors containing adipose tissue and normal hematopoietic cells that mainly occur in the adrenal cortex. Mediastinal myelolipomas are extremely rare. The three-dimensional, high-definition view with up to 10 times image magnification, 7 degrees of freedom of surgical instruments, and filtration of physiologic hand tremors with robotic systems are ideal for removing mediastinal tumors in a small space. Herein, we report a case of posterior mediastinal myelolipoma treated using robotic resection.

**Case presentation:**

A 72-year-old man with an abnormal shadow on a chest roentgenogram was referred to our hospital. Computed tomography revealed extrapulmonary paravertebral lesions in the right posterior mediastinum. Despite the tumor's fragility, complete resection was achieved with robot-assisted thoracic surgery while preserving the lesion integrity. The tumor was diagnosed as a myelolipoma using histopathology of the resected section, and the patient was discharged with no complications on the fifth postoperative day.

**Clinical discussion:**

Mediastinal myelolipomas are fragile tumors with a thin capsule, consisting of adipose tissue and normal hematopoietic cells. Robot-assisted thoracic surgery is particularly effective for resecting posterior mediastinal tumors like myelolipomas that contain adipose components.

**Conclusions:**

With delicate manipulation, robot-assisted thoracic surgery can be used to safely and completely resect fragile posterior mediastinal tumors, including myelolipomas.

## Highlights

• Myelolipomas are rare tumors consisting of adipose tissue and normal hematopoietic cells, typically found in the adrenal cortex, with mediastinal cases being extremely rare.
• Robot-assisted thoracic surgery enables precise and safe resection of fragile posterior mediastinal tumors like myelolipomas.

## 1 Introduction

Myelolipomas (MLs) are benign tumors that arise mainly in the adrenal cortex, and posterior mediastinum involvement is rare. MLs are fragile and can be easily damaged during surgical manipulation; however, robot-assisted thoracic surgery (RATS) with delicate handling facilitates resection without damage. Herein, we report a case of posterior mediastinal ML treated using robotic resection. This work has been reported in line with the Surgical CAse REport (SCARE) criteria [1].

## 2 Case presentation

**Fig. 1:**
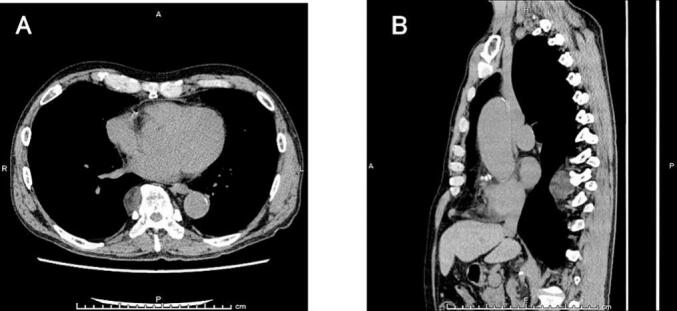
Chest CT image showing a well-encapsulated tumor 3 cm in diameter with a heterogeneous interior adjacent to the right side of the Th8 to Th10 thoracic vertebrae (A: axial mediastinal window, B: sagittal mediastinal window).

A 72-year-old man with a history of hypertension and nonvalvular atrial fibrillation was referred to our hospital with a suspected posterior mediastinal tumor. Chest computed tomography (CT) revealed a well-encapsulated tumor 3 cm in diameter with a heterogeneous interior adjacent to the right side of the 8th to 10th thoracic vertebrae (Fig. 1). Magnetic resonance imaging (MRI) could not be performed due to claustrophobia. Based on these imaging findings and localization, we suspected a neurogenic tumor and planned surgical excision using RATS.

**Fig. 2:**
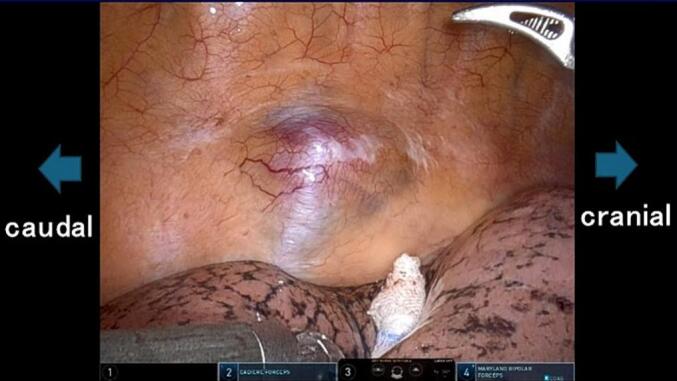
Operative findings. The tumor is well encapsulated and dark red presenting as a soft oval mass located in the posterior mediastinum bordering the Th9 to Th10 thoracic vertebrae.

**Fig. 3:**
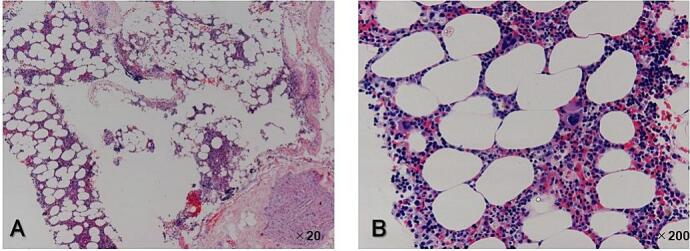
Histopathological findings revealed that the tumor was composed of mature adipocytes mixed with hematopoietic elements, including megakaryocytes and cells of regular granulopoietic and erythropoietic cell lineages (A: hematoxylin and eosin stain, original magnification ×20, B: ×200).

The patient was placed in a slightly forward tilt, left lateral decubitus position under general anesthesia. RATS was performed via the right intercostal approach using the da Vinci Xi system (Intuitive Surgical, Mountain View, CA, USA) with three arms. An 8 mm trocar was first inserted into the sixth intercostal space (ICS) along the middle axillary line. Another 8 mm trocar for the right arm was inserted along the posterior axillary line at the fourth ICS. A third 8 mm trocar for the left arm was inserted into the ninth ICS along the posterior axillary line. The three robotic trocars were placed in an inverted triangular configuration relative to the lesion, allowing precise instrument manipulation without collisions. After maintaining the pressure in the thoracic cavity at 10 mmHg, the da Vinci robot was docked. On the left, a Cadiere grasper was installed, while on the right, Maryland bipolar forceps was placed. The tumor was well encapsulated and dark red and presented as a soft oval mass located in the posterior mediastinum, bordering the Th9 to Th10 thoracic vertebrae (Fig. 2). The tumor was completely resected without damaging the capsules. The cut surface was yellowish with a homogeneous solid mass. No serious complications were observed during the postoperative course. The chest drain was removed on the first postoperative day, and the patient was discharged on the fifth postoperative day. Postoperative microscopic examination revealed predominantly mature adipose tissue with hematopoietic tissue comprising erythroblasts, megakaryocytes, and granulocytes (Fig. 3). Based on these findings, the patient was diagnosed with ML originating from the posterior mediastinum. He has been well for 3 years after surgery without recurrence.

## 3 Discussion

MLs are benign tumors composed of mature adipocytes and hematopoietic elements and were first described by Edgar von Gierke in 1905 and named by Charles Oberling in 1929 [2,3]. Eighty-five percent of MLs are of adrenal origin, while half of the non-adrenal MLs occur in the anterior sacral region, 13 % occur in the chest wall, and approximately 8 % occur in the mediastinum [4,5].

The origin of MLs is not yet well understood. Four hypotheses have been proposed to explain its pathogenesis: (i) MLs are derived from bone marrow emboli that lodge in the adrenal gland or other sites [6]. Several studies have suggested that metaplastic changes in embryonic primitive mesenchymal cells or embolisms of bone marrow cells occur in MLs via the bloodstream [7,8]. (ii) Chromosomal translocations identified in MLs cells [9] are the same karyotype disorders that are observed in benign lipomatous neoplasms. (iii) Most of these tumors are attached to vertebral bodies. Hematopoietic tissues may project from these sites to the paravertebral space, possibly through microfractures. Ectopic hematopoietic tissue can contain stem cells that induce MLs development [10]. (iv) The growth and variation of two cell types (ectopic adrenal and hematopoietic stem cells) are triggered by obesity, hypertension, chronic inflammation, or carcinoma, which have been studied in some reported cases [11,12].

MLs can present with nonspecific symptoms, including cough or chest pain, but most cases are asymptomatic and are discovered incidentally during the follow-up of other diseases or medical check-ups.

Imaging modalities, including CT and MRI are used for MLs diagnosis. CT reveals a well-defined tumor with heterogeneities based on its composition with myeloid elements having high attenuation values and fat tissue having low attenuation. In contrast-enhanced CT, MLs, due to their low vascularity, exhibit minimal to no enhancement MRI shows isointensity or slight hyperintensity on T1- and T2-weighted images. After the application of contrast media, myeloid elements show moderate enhancement, while adipose tissue shows no enhancement, resulting in heterogeneous enhancement [13]. Although CT and MRI are effective in diagnosing myelolipoma, the imaging differential diagnoses for fat-containing lesions are often extensive and include nonneoplastic, benign, and malignant entities. Thus, a definitive diagnosis of MLs is difficult to establish by imaging alone.

Among posterior mediastinal tumors, the most commonly differential diagnosis is a malignant retropleural fat-containing tumor (liposarcoma). Myelolipomas tend to have clear margins in contrast to liposarcomas, which tend to be less well-circumscribed and vary according to the subtypes. Well differentiated liposarcoma typically contains more than 75 % adipose tissue, and dedifferentiated liposarcoma can be quite complex on imaging, often containing heterogeneous nonlipomatous components.

Other differential diagnoses include extramedullary hematopoiesis, tumor, lymphoma, and neurogenic teratoma.

FDG-PET/CT findings can vary depending on the composition of the tumor. Low or absent FDG uptake (SUVmax near background fat) in fat-rich myelolipomas. Mild to moderate FDG uptake in hematopoietic-rich myelolipomas, due to active bone marrow elements. Heterogeneous FDG uptake if there is a mix of fat and hematopoietic tissue.

Most reported mediastinal MLs occur in the posterior mediastinum, with only a few cases of anterior mediastinal MLs reported [14,15]. In patients with posterior mediastinal origin, tumor differentiation often involves suggestive imaging findings of adipose-containing tumors, including neurogenic tumors, lymphomas, lipomas, well-differentiated liposarcomas, and extramedullary hematopoiesis, with mixed radiological features of fatty components. However, differentiating MLs from extramedullary hematopoiesis is particularly challenging, as both present similar histological findings with a mixture of mature fat cells and hematopoietic cells on both CT and MRI [16]. The former often presents as a solitary case and is frequently associated with comorbidities including obesity, hypertension, and diabetes, while the latter occurs in multiple cases and is characterized by anemia, splenomegaly, and histopathological features of lymphoid aggregates [17].

Mediastinal MLs treatment typically involves either observation or surgery; however, no clear consensus has been established on specific size criteria for either approach. Nonetheless, surgical resection is favored in most cases due to the potential increase in tumor size in the future and the difficulty of preoperative diagnosis. Surgery should also be considered if symptoms such as coughing, wheezing, or nerve compression are present. However, MLs are considered benign tumors, and no studies on malignant transformation have been reported thus far. Therefore, in patients with a confirmed diagnosis, observation can be considered an option, especially for patients with small and asymptomatic tumors. The surgical approach for mediastinal tumors is determined by the tumor size and location. Although video-assisted thoracoscopic surgery (VATS) is a well-established minimally invasive approach for accessing mediastinal tumors, with the recent developments in surgical instruments, robot-assisted thoracic surgery has an increasing usage throughout the world. Comparing with VATS, RATS offers superiorities, in terms of tremor filtration, three-dimensional visualization, ten-times-enlarged image and seven-degree freedom of its dexterity endowrists. RATS has already widely used in lobectomy and other minimally invasive thoracic surgeries in recent years.

In the report of comparison of postoperative outcomes after multiportal RATS (M-RATS) and uniportal VATS (U-VATS) for non-small cell lung cancer, M-RATS and U-VATS achieved comparable symptom burden and functional impairment after discharge [18]. Compared to U-VATS, M-RATS caused less intraoperative blood loss, and more stations of dissections of dissected lymph nodes, more numbers of dissected lymph nodes, but was associated with more severe pain and activity limitation in the short postoperative period [18].

Comparative studies of VATS and RATS for posterior mediastinal neurogenic tumors show that RATS has superiorities in terms of surgical blood loss and postoperative hospital stay over VATS, and the authors of this report conclude that RATS could be a feasible and safe way for resecting posterior mediastinal neurogenic tumor [19]. However, it is essential to acknowledge that the RATS technique also has certain disadvantages, including longer setup times (which improve markedly with operating room experience) and longer intraoperative instrument change intervals. Additionally, the acquisition of new robotic instruments is costlier [20].

As previously mentioned, while RATS has certain drawbacks compared to VATS, it enables more delicate and precise surgical maneuvers. This makes RATS a potentially superior approach to VATS for fragile lesions, such as mediastinal MLs.

## 4 Conclusion

Here, we present a case in which a posterior mediastinal ML was successfully resected using RATS. RATS can be used for the safe and complete resection of fragile posterior mediastinal tumors, such as MLs by facilitating delicate manipulation.

## Consent

Written informed consent was obtained from the patient for publication of this case report and accompanying images. A copy of the written consent is available for review by the Editor-in-Chief of this journal on request.

## Ethical approval

Case reports are exempt from the “Ethical Guidelines for Life Sciences and Medical Research Involving Human Subjects” and do not require approval by the IRB of our institution.

## Guarantor

Kiyoshi Sato

## Research registration number

N/A

## Funding

Not applicable.

## Author contribution

K.S. wrote the whole of the manuscript and took responsibility for the construction of the whole or body of the manuscript. S.Y., K.T., S.F., and N.H. took in data management. T.K. organized and supervised the article. All authors have read and approved the final manuscript.

## Conflict of interest statement

The authors declare that they have no competing interests.
